# High-Resolution Ultrasound Evaluation of Common Dorsal Hand Rejuvenation Techniques: A Multicenter Study

**DOI:** 10.7759/cureus.101017

**Published:** 2026-01-07

**Authors:** Claudia Gonzalez, Valeria Duque-Clavijo, Sandra Suárez, Ernesto Barbosa, Alejandro Coello, Isaac Shturman, Tatiana Delvasto

**Affiliations:** 1 Radiology, Rosario University, Bogota, COL; 2 Medicine, Universidad de los Andes, Bogota, COL; 3 Aesthetic Medicine, Grupo Presenza, Bogota, COL; 4 Plastic Surgery, Barma Functional and Aesthetic Medicine, Bogota, COL; 5 Facial and Body Aesthetics, Xtetic Clinique by Dr. Alejandro Coello, Mexico City, MEX; 6 Plastic Surgery, Plastic Shturman at Angeles Lomas Hospital, Mexico City, MEX; 7 Radiology, Private Practice, Medellin, COL

**Keywords:** calcium hydroxylapatite, fat transplantation, hand rejuvenation, high-resolution ultrasound with doppler, hyaluronic acid

## Abstract

Background

Hand dorsum volumization is an increasingly used aesthetic procedure, but the ultrasonographic appearance of commonly injected materials has not been systematically described. High-resolution ultrasound with Doppler (HRUD) may provide an objective method to verify product placement and support safer, standardized hand rejuvenation. This descriptive study aimed to characterize the postprocedural high-resolution ultrasonographic features of dorsal hand volumization using commonly employed fillers and autologous fat grafting.

Methods

We conducted a multicenter, descriptive study at three aesthetic medicine centers in Bogotá, Colombia, and Mexico City, Mexico, between March and June 2025. Adults requesting dorsal hand volumization received hyaluronic acid, calcium hydroxylapatite (CaHA), a poly-L-lactic acid (PLLA)-hyaluronic acid mixture, a factory-prepared CaHA-hyaluronic acid hybrid filler, or autologous fat grafting. All products were injected into the subdermal plane using a standardized cannula technique. HRUD was performed at baseline, early post-procedure, and at follow-up. Baseline aging was graded using the Merz Hand Grading Scale, and outcomes were assessed at two months using the Modified Aesthetic Response Scale (MARS).

Results

Twelve patients were included (mean age, 56 years; range, 44-66 years; 11 women). HRUD enabled clear identification of dorsal hand anatomy and material-specific sonographic patterns: CaHA appeared as hyperechoic cloud-like bands, hybrid fillers as lobulated hyperechoic structures, hyaluronic acid as hypoechoic pseudocystic areas, PLLA-hyaluronic acid mixtures as diffuse echogenicity with linear bands, and fat grafts as elongated isoechoic structures. In all cases, injected material was located superficial to the extensor tendons, with transient postinjection vascularization observed on Doppler. No early or late complications were detected. At two months, most patients demonstrated favorable short-term aesthetic improvement.

Conclusion

HRUD provides a practical method for documenting dorsal hand anatomy, confirming correct subdermal placement of volumizing materials, and recognizing distinct sonographic signatures. Its use may assist experienced injectors in enhancing procedural safety and monitoring. Given the short follow-up, findings should be interpreted cautiously, and larger studies with longer follow-up are warranted.

## Introduction

Hand dorsum volumization has become an increasingly relevant aesthetic procedure for restoring a youthful hand appearance, as the hands represent a key indicator of perceived age due to their high visibility and the characteristic anatomical changes associated with aging, including volume loss, skin thinning, and increased prominence of veins and tendons [1,2]. Various minimally invasive techniques, used alone or in combination, can achieve natural, durable results. The most commonly used products include dermal fillers and autologous fat grafts. However, despite the growing adoption of these procedures, few studies have comprehensively described the ultrasonographic characteristics of different filler materials in the dorsal hand, leaving the need for a standardized, imaging-based approach to evaluation.

Although high-resolution ultrasound has been increasingly incorporated into aesthetic practice, most published reports focus on isolated techniques or single-center experiences. The present study provides a multicenter, systematic application of high-resolution ultrasound with Doppler (HRUD) for the evaluation of dorsal hand rejuvenation using multiple volumization approaches, including different filler materials and autologous fat grafting. By integrating standardized pre- and post-procedural ultrasound assessment, this work highlights the clinical value of HRUD in objective anatomical evaluation, confirmation of product placement, and early detection of procedure-related changes.

The objective of this descriptive study was to describe the postprocedural ultrasonographic characteristics of the dorsal hand following volumization with four filler types: hyaluronic acid, calcium hydroxylapatite (CaHA), a mixture of poly-L-lactic acid (PLLA) and hyaluronic acid, and a factory-prepared hybrid filler composed of CaHA and hyaluronic acid, as well as with autologous fat grafting. We used HRUD to characterize each filler’s sonographic appearance, anatomical distribution, degree of volumetric restoration, and any associated vascular changes.

## Materials and methods

Study design and participants

This multicenter, descriptive study was conducted at three aesthetic medicine centers: one in Bogotá, Colombia (Highly Specialized Ultrasound Center), and two in Mexico City, Mexico (Xtetic Clinique by Dr. Alejandro Coello and Plastic Shturman at Ángeles Lomas Hospital). Data collection was performed over two months, from March 1 to June 1, 2025. Twelve voluntary patients seeking dorsal hand volumization for aesthetic hand rejuvenation were recruited.

Follow-up was standardized across all study centers. All patients underwent baseline clinical and high-resolution ultrasound evaluation prior to treatment, a repeat clinical and ultrasound assessment on the day immediately following the procedure, and a final standardized clinical and ultrasound evaluation two months after treatment.

Inclusion and exclusion criteria

Adults of both sexes aged 44 to 66 years who requested dorsal hand volumization for aesthetic purposes and demonstrated objective clinical evidence of aging changes were included. Clinical assessment of eligibility incorporated the Merz Hand Grading Scale and ultrasound-based evaluation of subcutaneous fat atrophy.

Patients were excluded if they had any medical contraindications to filler use, a history of allergy or hypersensitivity to fillers or lidocaine, active infection or skin lesions at or near the injection site, uncontrolled acute or chronic inflammatory hand conditions, or lymphedema.

Ethical considerations

The study protocol was reviewed and approved by the institutional Research Ethics and Bioethics Committees (approval numbers: CEB-1-2025, CEB-2-2025, and CEB-3-2025). All procedures were performed in accordance with the ethical principles of the Declaration of Helsinki. Verbal informed consent for the ultrasonographic evaluation and associated aesthetic procedures was obtained from all participants and documented in their clinical records, as approved by the ethics committees, given the nature of the study. All patient data were anonymized and stored securely with restricted access to preserve confidentiality. Participants were fully informed about the purpose, scope, and data handling measures of the study, ensuring complete adherence to ethical standards.

Training and expertise of professionals

The study was conducted across three centers with clearly defined roles for each professional. In Bogotá, Colombia, all HRUD examinations were performed at a specialized dermatologic ultrasound center by a radiologist with more than 15 years of experience in dermatologic and aesthetic ultrasound. Injectable procedures in Bogotá were performed by one board-certified plastic surgeon and one aesthetic medicine physician, both with more than 15 years of experience in injectable aesthetic treatments.

In Mexico City, procedures were carried out at two centers: Xtetic Clinique and Ángeles Lomas Hospital. At each site, the treating physician (Dr. Alejandro Coello at Xtetic Clinique and Dr. Isacc Shturman at Ángeles Lomas Hospital) performed both the HRUD examinations and the injectable procedures. Both physicians are certified in high-resolution ultrasound and have more than 15 years of experience in injectable aesthetic procedures.

Aesthetic ultrasound evaluation

HRUD was used as the primary diagnostic tool for the detailed evaluation of dorsal hand anatomy. All examinations were performed using the same ultrasound equipment across all study sites, specifically a General Electric Venue™ system (Cincinnati, OH, USA) equipped with a linear multi-resolution hockey-stick transducer operating at 8-18 MHz, in accordance with previously published guidelines [3,4]. Grayscale, color Doppler, and duplex ultrasound assessments were conducted both before and after the volumization procedures. All devices employed the factory preset known as “DERMA,” which standardizes grayscale and color Doppler parameters for optimal visualization of skin and subcutaneous tissues in aesthetic and dermatologic applications.

HRUD image acquisition was performed locally at each study site. In Bogotá, Colombia, all ultrasound examinations were conducted at a specialized dermatologic ultrasound center by a radiologist with more than 15 years of experience in dermatologic and aesthetic ultrasound. In Mexico City, HRUD image acquisition was performed by the treating physicians, both of whom hold formal certification in high-resolution ultrasound.

To ensure consistency and standardization across centers, all ultrasound images obtained outside Bogotá were subsequently reviewed and reinterpreted by the same expert radiologist to verify technical quality, adherence to standardized acquisition protocols, and consistency of image interpretation.

The severity of dorsal hand subcutaneous fat atrophy was assessed using grayscale HRUD and classified according to the proportional reduction in the thickness of the subcutaneous fat layer. Findings were categorized as normal, defined as no detectable thinning of the subcutaneous fat layer; mild, defined as an approximate reduction of one-third of the normal fat thickness; moderate, defined as an approximate reduction of two-thirds of the normal fat thickness; and severe, defined as near-complete or complete loss of the subcutaneous fat layer.

Color Doppler activity was evaluated using a semi-quantitative grading system based on capillary density within a standardized area of interest. Mild hyperemia was defined as the presence of up to four capillaries per square centimeter of examined tissue, moderate hyperemia as five to seven capillaries per square centimeter, and marked hyperemia as more than eight capillaries per square centimeter.

Injection procedure

The selection of the product for hand dorsum volumization was guided by patient-specific anatomical deficits and treatment goals, as well as the established safety and efficacy profiles of available products for dorsal hand rejuvenation. Injector expertise was considered essential to ensure appropriate and safe application. All injectable products used in this study contained integrated lidocaine; therefore, no additional topical anesthesia, local anesthetic infiltration, or sedation was required for filler-based procedures. Regardless of the product selected, a standardized injection technique was applied in all patients, consisting of a single dorsal entry point, placement of the product in the subdermal plane using a cannula with a retrograde threading technique, followed by gentle digital massage to promote homogeneous distribution throughout the dorsal hand.

Volumization With Hyaluronic Acid

For patients treated with hyaluronic acid filler, 1 mL of highly cross-linked hyaluronic acid was injected into the dorsal aspect of each hand using a 22-G × 70-mm cannula.

Volumization With CaHA

For patients treated with CaHA, a mixture of 1.5 mL of CaHA and 1.5 mL of lidocaine without epinephrine was prepared, and 1.5 mL of this mixture was injected into the dorsum of each hand using a 22G × 70-mm cannula.

Volumization With PLLA and Hyaluronic Acid

For patients treated with PLLA, one vial of PLLA was reconstituted with 8 mL of normal saline solution (NSS) and 2 mL of lidocaine without epinephrine; 5 mL of this mixture was then injected into the dorsum of each hand using a 22G × 70-mm cannula, after which 1 mL of hyaluronic acid was injected into each hand using a 30G × 13-mm needle.

Volumization With Hybrid Calcium Hydroxyapatite and Hyaluronic Acid

For patients treated with the factory-prepared CaHA-hyaluronic acid hybrid filler (HarmonyCa®, Allergan Aesthetics, an AbbVie company, Irvine, CA, USA; CaHA-hyaluronic acid hybrid filler), 1.25 mL of undiluted product was injected into the dorsum of each hand using a 22G × 70-mm cannula.

Volumization With Autologous Fat Grafting

A 55-year-old patient underwent liposuction under general anesthesia. A tumescent solution containing 1,000 mL of NSS and 1 mL of epinephrine per 1,000 mL was prepared. Tumescent infiltration was performed in the lower abdominal quadrants, which served as the exclusive donor site for fat harvesting. Liposuction was performed using 4-mm cannulas. The harvested fat was decanted and filtered through a sterile mesh, after which 600 mg of clindamycin was added to the isolated adipose tissue. Finally, 3 mL of processed fat was injected into the dorsum of each hand using a 1-mm cannula.

Pre- and post-procedure clinical and HRUD evaluation

Preprocedural HRUD of the dorsal hand was performed to rule out the presence of preexisting fillers, identify the different anatomical layers, and assess the degree of fatty tissue atrophy, prominence of the superficial venous plexus, and visibility of the extensor tendons. Color and duplex Doppler imaging were also used to evaluate slow venous flow within the superficial plexus and to exclude subclinical inflammatory activity in any dorsal hand layer. One day after the volumization procedure, HRUD was repeated to verify the uniformity of product distribution and to confirm correct anatomical localization. Doppler analysis was also performed to confirm satisfactory vascular flow and to rule out early complications, such as hematoma or seroma.

A comprehensive physical examination was performed before the procedure to establish a baseline assessment. The Merz Hand Grading Scale was used to classify the degree of dorsal hand aging (Table 1), as originally proposed and validated by Carruthers et al [5]. A clinical evaluation was conducted immediately after the procedure to identify early adverse events, and a second evaluation was performed the following day, at the time of the HRUD examination, to rule out early postprocedural complications.

**Table 1:** Merz Hand Grading Scale for assessment of dorsal hand aging Reproduced from [1] under the terms of the Creative Commons Attribution 4.0 International License (CC BY 4.0). Original source for the Merz Hand Grading Scale: [5]

Grade	Clinical Findings
0	Normal skin, no loss of fatty tissue
1	Mild loss of fatty tissue, slight visibility of veins
2	Moderate loss of fatty tissue, mild visibility of veins and tendons
3	Severe loss of fatty tissue, moderate visibility of veins and tendons
4	Extreme loss of fatty tissue, severe visibility of veins and tendons

Follow-up and aesthetic outcome assessment:

Two months after the procedure, the treating physician conducted a follow-up visit to exclude late complications and to assess patient satisfaction and overall aesthetic outcome. For this final evaluation, the Modified Aesthetic Response Scale (MARS) was used. This five-point ordinal scale was developed by the authors to qualitatively assess global aesthetic improvement after hand rejuvenation and was conceptually adapted from the Global Aesthetic Improvement Scale [6]. The MARS categories and descriptors are presented in Table 2.

**Table 2:** Modified Aesthetic Response Scale The Modified Aesthetic Response Scale was designed by the authors to assess overall perceived aesthetic improvement after hand rejuvenation, conceptually adapted from the Global Aesthetic Improvement Scale [6].

Rating	Level of Improvement	Description
5	Excellent enhancement	Marked improvement in global appearance with clear rejuvenation and restoration of hand contour.
4	Noticeable improvement	Visible aesthetic benefit with moderate enhancement in texture and tone.
3	Mild improvement	Subtle but perceptible positive change compared to baseline.
2	No evident change	No observable difference relative to baseline.
1	Deterioration	Worsening of the overall appearance compared to baseline.

Statistical analysis

Data were analyzed using descriptive statistical methods, with spreadsheet-based analysis performed in Microsoft Excel (Microsoft Corp, Redmond, WA, USA). Continuous variables are summarized as medians with ranges, and categorical variables as frequencies and percentages. Given the limited sample size and exploratory design of this case series, all comparisons between volumization techniques were qualitative and descriptive, and no inferential statistical testing was performed.

## Results

**Figure 1:**
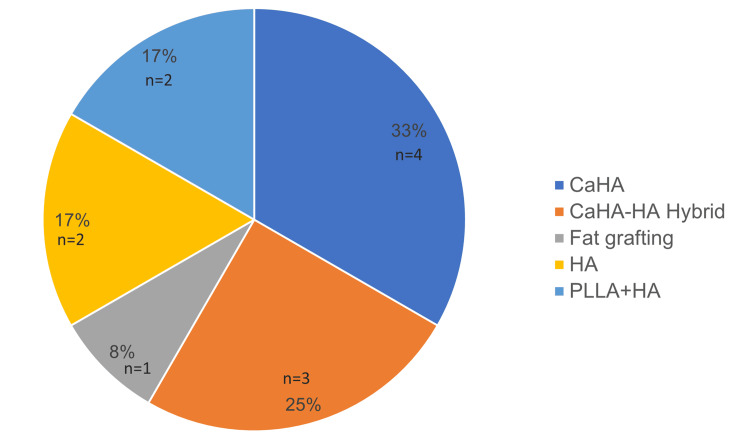
Distribution of products used for hand rejuvenation Abbreviations: CaHA, calcium hydroxylapatite; CaHA–HA hybrid (HarmonyCa), factory-prepared calcium hydroxylapatite–hyaluronic acid hybrid filler; HA, hyaluronic acid; PLLA+HA, poly-L-lactic acid plus hyaluronic acid.

A total of 12 patients were included in the study. The mean age was 56 years (range, 44-66 years). Of the total sample, one patient was male, and the remaining patients were female. Based on the Merz Hand Grading Scale, 33.3% (n = 4) of patients were classified as grade 2, 50% (n = 6) as grade 3, and 16.6% (n = 2) as grade 4. The most frequently used product for rejuvenation was CaHA filler (Radiesse®; Merz Aesthetics, Raleigh, NC, USA), which was applied in 33.3% (n = 4) of patients. This was followed by the factory-prepared hybrid CaHA-hyaluronic acid filler (HarmonyCa®) in 25% of patients (n = 3). Only one patient (8.3%) underwent autologous fat grafting (Figure 1).

**Figure 2:**
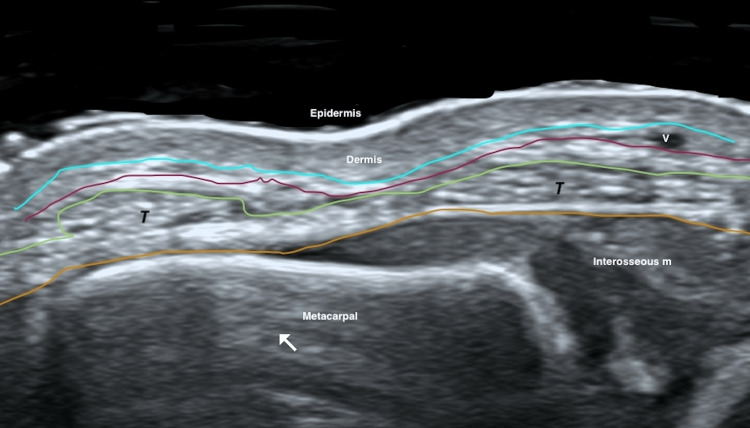
HRUD of normal dorsal hand anatomy (transverse view) Color overlays delineate key fascial planes and structures: aquamarine line, superficial dermal fascia; purple line, superficial venous fascia; green line, intermediate dorsal fascia; yellow line, deep dorsal fascia. The superficial venous plexus (v), extensor tendons (T), and metacarpal bone (white arrow) are indicated. Abbreviations: HRUD, high-resolution ultrasound with Doppler; v, superficial venous plexus; T, extensor tendons.

HRUD clearly delineated the normal anatomical layers of the dorsal hand in all patients (Figure 2).

**Figure 3:**
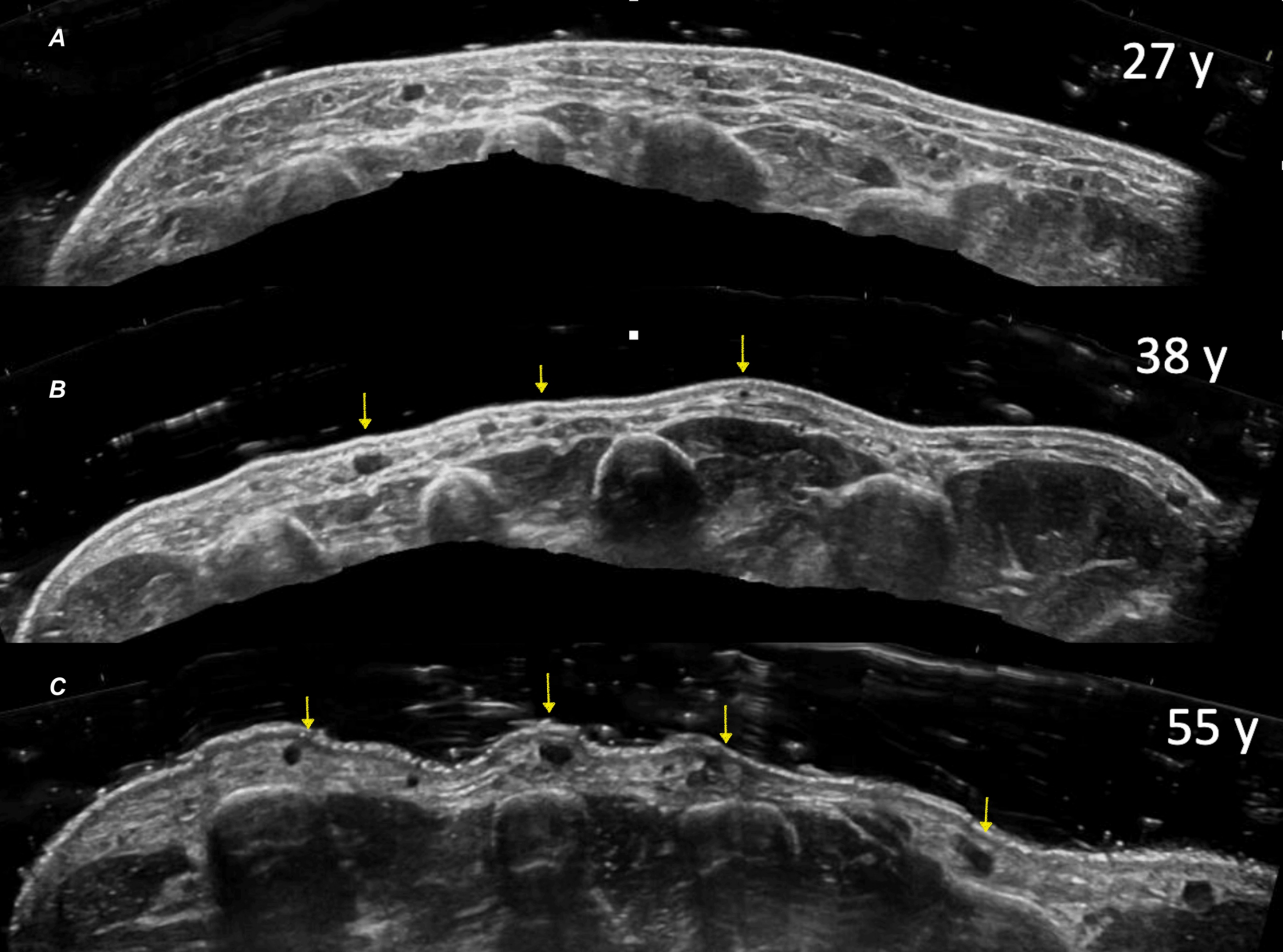
HRUD of dorsal hand aging patterns across the adult lifespan (transverse views) (3A) Woman aged 27 years with preserved subcutaneous fat and normal dorsal hand anatomy; (3B) Woman aged 38 years with moderate fat loss; yellow arrows highlight mild prominence of the dorsal venous plexus; (3C) Woman aged 55 years with marked fat loss; yellow arrows indicate pronounced venous plexus prominence. Abbreviations: HRUD, high-resolution ultrasound with Doppler.

Preprocedure HRUD showed that six patients (50%) had severe loss of fatty tissue with moderate visibility of veins and extensor tendons. An additional four patients (33.3%) had moderate loss of fatty tissue and mild visibility of veins and tendons. The remaining two (16.6%) patients demonstrated extreme loss of fatty tissue with severe visibility of veins and tendons (Figure 3).

**Figure 4:**
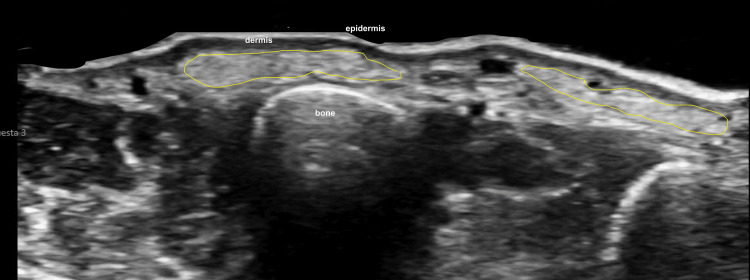
Sonographic appearance of CaHA filler in the dorsal hand (transverse view) Yellow boundary lines outline well-defined hyperechoic bands with a characteristic “cloud-like” configuration corresponding to CaHA deposits (Radiesse®; Merz Aesthetics, Raleigh, NC, USA). Abbreviations: CaHA, calcium hydroxylapatite.

HRUD analysis allowed precise identification, differentiation, and characterization of each filler material in all patients. In patients treated with CaHA filler (Radiesse®), the immediate postprocedural sonographic pattern consisted of well-defined hyperechoic bands with a characteristic “cloud-like” appearance (Figure 4). This appearance was consistent across all CaHA-treated patients and was easily distinguishable from the surrounding soft tissues.

**Figure 5:**
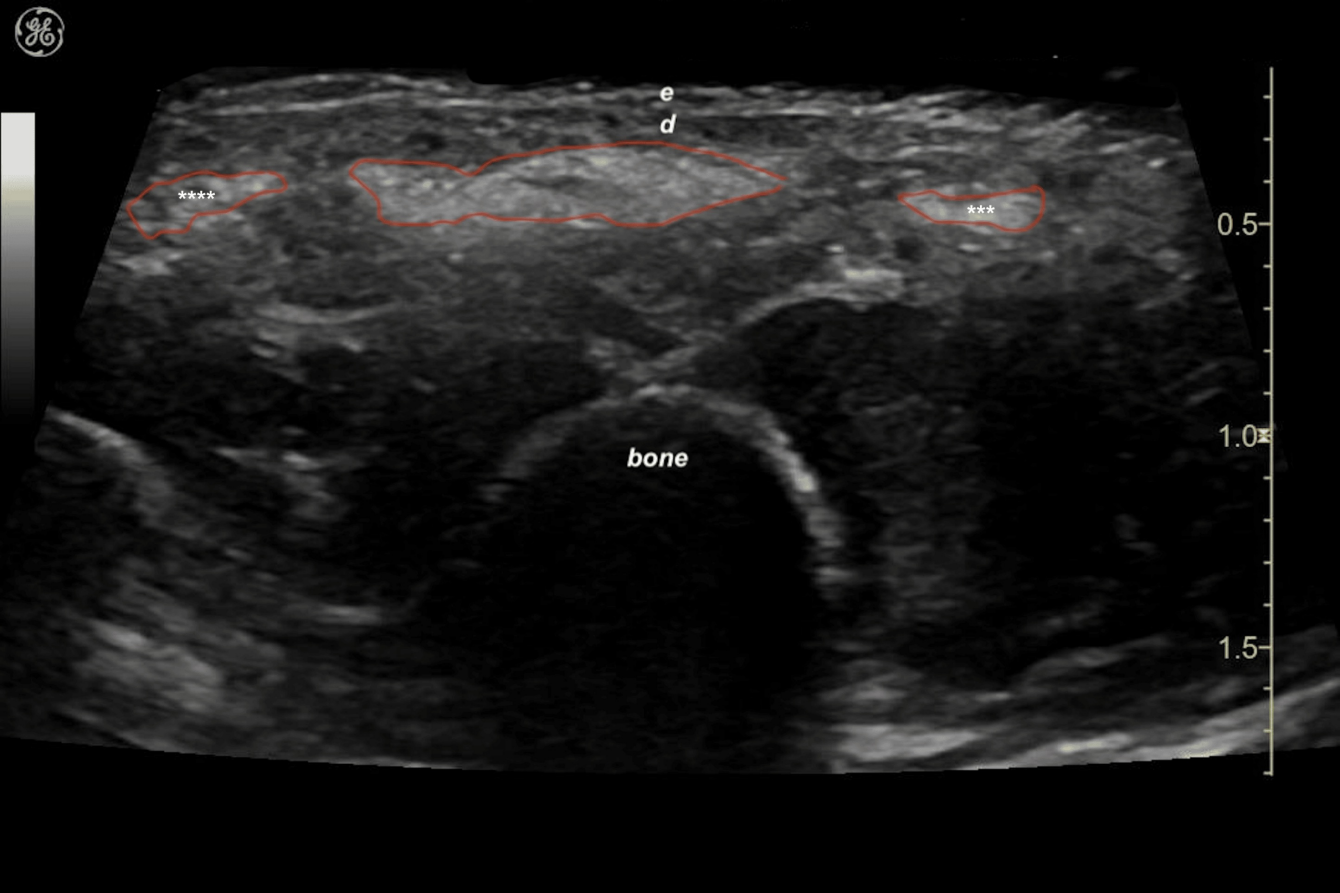
Sonographic appearance of factory-prepared CaHA–hyaluronic acid hybrid filler (HarmonyCa®) in the dorsal hand (transverse view) Red boundary lines outline hyperechoic deposits with echogenicity and band-like distribution similar to CaHA alone, but with more lobulated contours; isolated globular foci are marked by asterisks (*). Epidermis (e) and dermis (d) are labeled. Product shown: HarmonyCa® (Allergan Aesthetics, an AbbVie company, Irvine, CA, USA). Abbreviations: CaHA, calcium hydroxylapatite; HA, hyaluronic acid

When CaHA was combined with the factory-prepared hyaluronic acid component in the hybrid filler (HarmonyCa®), the ultrasonographic deposits showed a pattern similar to CaHA alone, but with a slightly more lobulated or undulating morphology (Figure 5).

**Figure 6:**
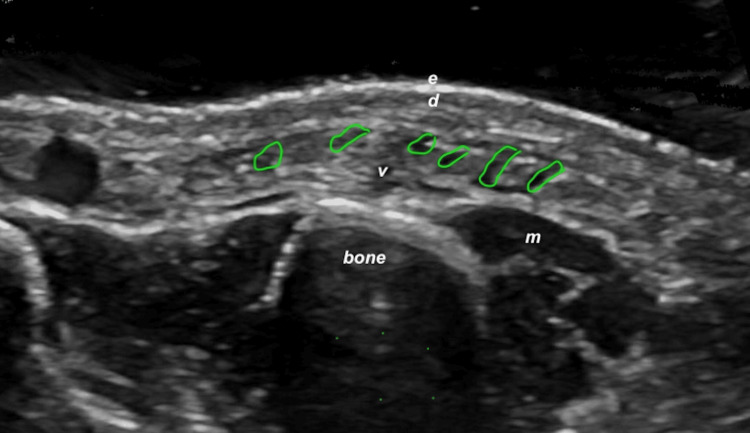
Sonographic appearance of HA filler in the dorsal hand (transverse view) Green boundary lines delineate multiple HA deposits with a characteristic pseudocystic, hypoechoic, laminar morphology. Epidermis (e), dermis (d), muscle (m), and bone (b) are labeled. Abbreviations: HA, hyaluronic acid

Hyaluronic acid alone, owing to its lower density, showed a typical pseudocystic hypoechoic appearance on ultrasound, often elongated and laminar in configuration (Figure 6). These sonographic differences facilitated material-specific recognition in the dorsal hand.

**Figure 7:**
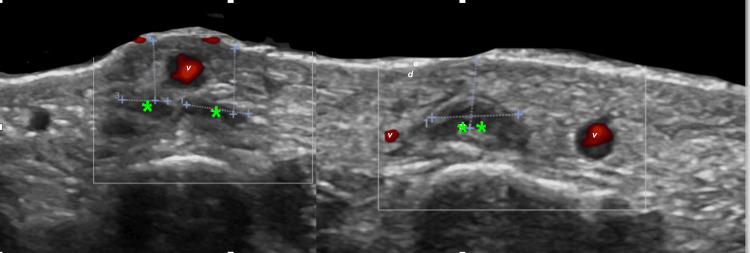
Sonographic appearance after combined PLLA and HA injection (transverse view) (A, B) Green asterisks (*) mark pseudocystic, hypoechoic HA deposits. Horizontal and vertical purple calipers (+) show transverse diameter and depth from the epidermal surface, respectively. Epidermis (e), dermis (d), and the superficial venous plexus (v) are labeled. Abbreviations: PLLA, poly-L-lactic acid; HA, hyaluronic acid

In two patients, volumization was performed with a mixture of hyaluronic acid and PLLA. Postprocedural ultrasound in these patients revealed a subtle and diffuse increase in echogenicity within the subcutaneous fat layer. Because this formulation required a higher dilution volume (10 cc of NSS), the increase in the thickness of the second layer on ultrasound was greater than that observed with the other fillers. In addition, hypoechoic linear bands corresponding to the NSS component were visible and ran parallel to the skin surface (Figure 7).

**Figure 8:**
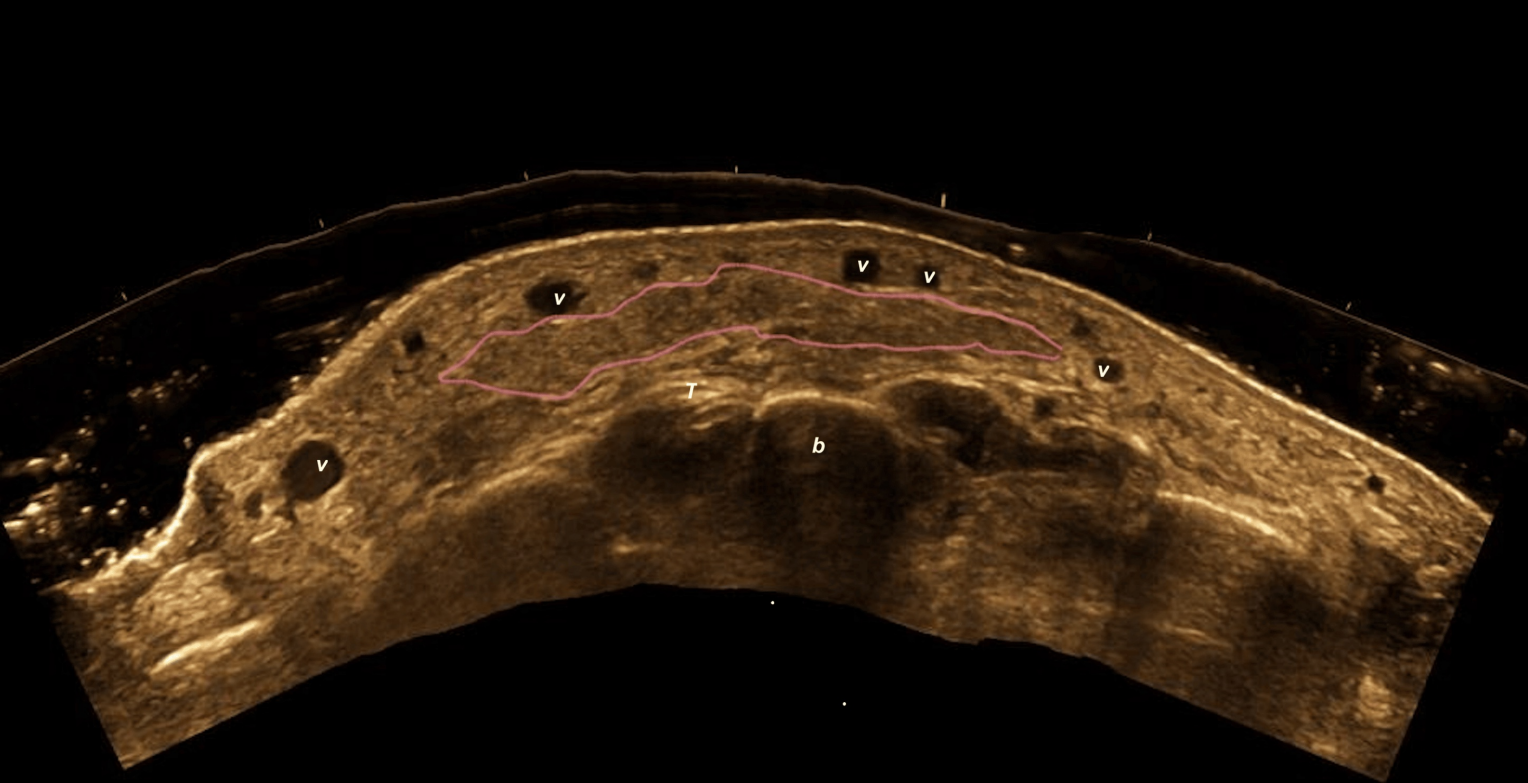
Sonographic appearance of autologous fat grafting in the dorsal hand (transverse view) Pink boundary lines outline elongated, isoechoic adipose deposits consistent with transplanted autologous fat. The superficial venous plexus (v), extensor tendon (T), and bone (b) are labeled.

Autologous fat grafting produced isoechoic images compared with native subcutaneous fat, with elongated deposits consistent with the morphology of transplanted adipose tissue (Figure 8).

**Figure 9:**
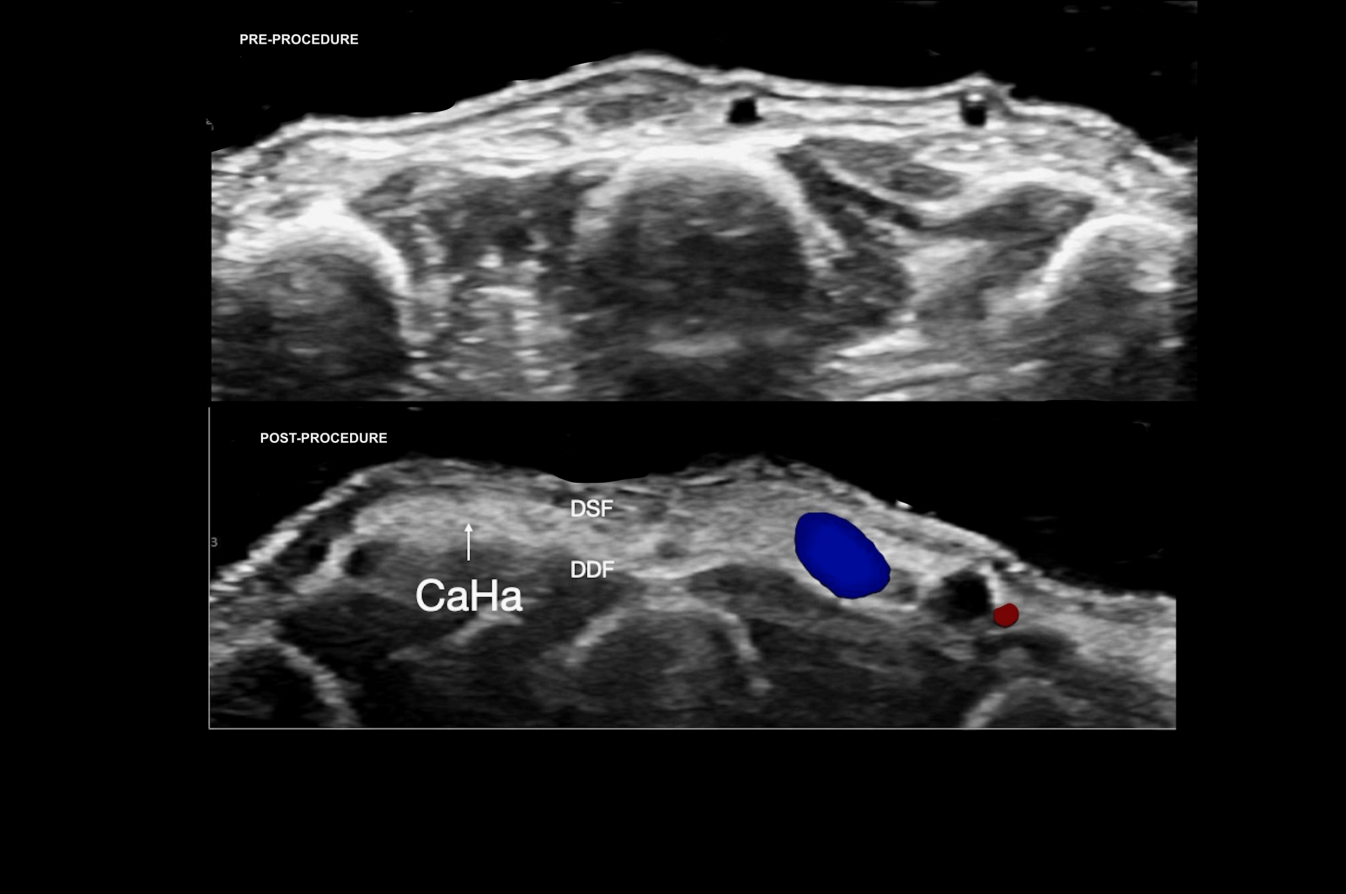
Pre- and postprocedure HRUD of dorsal hand volumization with CaHA (transverse views) (A) Preprocedure image demonstrates atrophic subcutaneous fat with prominent dorsal venous plexus (yellow arrows). (B) Postprocedure image shows hyperechoic, band-like CaHA deposits (white arrow) situated between the SDF and DDF, restoring dorsal hand volume. Filler shown: CaHA (Radiesse®; Merz Aesthetics, Raleigh, NC, USA). Abbreviations: HRUD, high-resolution ultrasound with Doppler; CaHA, calcium hydroxylapatite; SDF, superficial dorsal fascia; DDF, deep dorsal fascia.

Ultrasound assessment confirmed that, in 100% (n = 12) of procedures, the injected material was deposited superficial to the extensor tendons, corresponding to the target aesthetic plane for volumization (Figure 9).

**Figure 10:**
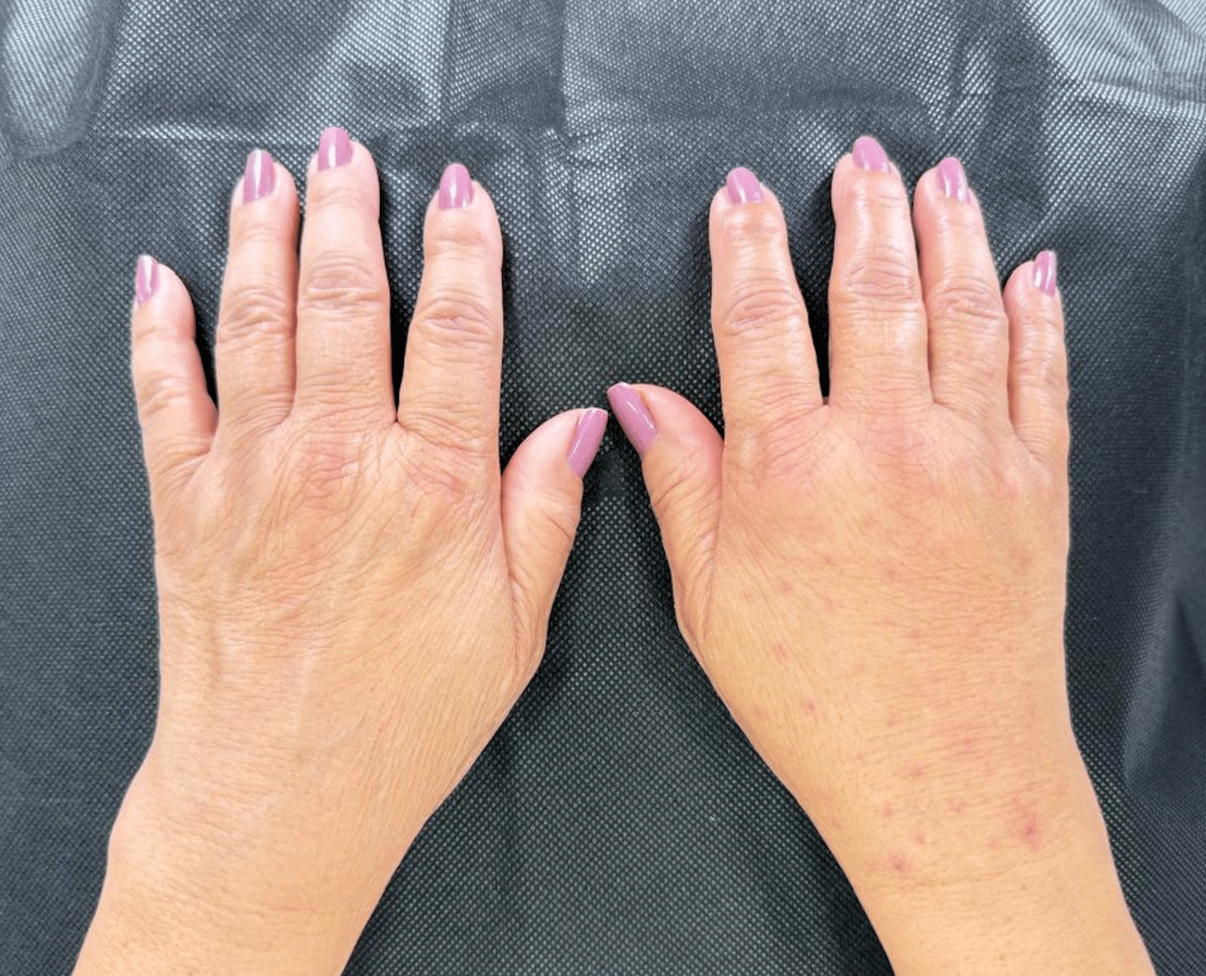
Clinical comparison of hands before and after volumization (A) Left hand, untreated with atrophic changes corresponding to Merz grade 3. (B) Right hand, same patient after combined PLLA and HA treatment shows restoration of dorsal hand volume and global rejuvenation changes. Abbreviations: PLLA, poly-L-lactic acid; HA, hyaluronic acid.

Doppler evaluation performed immediately after the procedure demonstrated increased vascularization in 100% (n = 12) of patients, which was interpreted as a transient postinjection change. No early or late complications, including hematoma, seroma, nodularity, or vascular compromise, were observed in any patient during the study period (Figure 10).

Two months after the procedure, the attending physician evaluated overall outcomes using the MARS. A total of 83.3% (n = 10) of patients were rated as score 5 (excellent enhancement), and 16.7% (n = 2) were rated as score 4 (noticeable improvement). These findings indicated a uniformly favorable aesthetic response across all product types. The complete set of clinical and ultrasonographic findings is summarized in Table 3.

**Table 3:** Patient characteristics, ultrasound findings, treatments, and short-term outcomes Abbreviations: F, female; M: male; HRUD, high-resolution ultrasound with Doppler; CaHA, calcium hydroxylapatite; HA, hyaluronic acid; PLLA, poly-L-lactic acid; PLLA+HA, poly-L-lactic acid plus hyaluronic acid; NSS, normal saline solution; MARS, Modified Aesthetic Response Scale. “+”, “++”, “+++” indicate relative Doppler hyperemia (mild, moderate, marked, respectively). Radiesse® (calcium hydroxylapatite; Merz Aesthetics, Raleigh, NC, USA); HarmonyCa® (calcium hydroxylapatite–hyaluronic acid hybrid filler; Allergan Aesthetics, an AbbVie company, Irvine, CA, USA). MARS scores: 5 = Excellent enhancement; 4 = Noticeable improvement; 3 = Mild improvement; 2 = No evident change; 1 = Deterioration.

Age (years)	Sex	Merz grade (pre)	HRUD preprocedure	Product	Dose per hand (as administered)	HRUD postprocedure (material pattern)	Location on HRUD (post)	Doppler (post)	Early complications	Late complications	MARS
64	F	3	Severe fat loss; moderate vein/tendon visibility	CaHA (Radiesse®)	1.5 mL CaHA + 1.5 mL lidocaine; 1.5 mL injected	Hyperechoic band-like CaHA deposits; no acoustic shadowing	Superficial to extensor tendons	++	None	None	5
56	F	3	Severe fat loss; moderate vein/tendon visibility	CaHA (Radiesse®)	1.5 mL CaHA + 1.5 mL lidocaine; 1.5 mL injected	Hyperechoic band-like CaHA deposits; no acoustic shadowing	Superficial to extensor tendons	+++	None	None	5
45	F	4	Extreme fat loss; severe vein/tendon visibility	Autologous fat	2 mL injected	Elongated, isoechoic adipose deposits	Superficial to extensor tendons	+	None	None	5
57	M	2	Moderate fat loss; mild vein/tendon visibility	CaHA (Radiesse®)	1.5 mL CaHA + 1.5 mL lidocaine; 1.5 mL injected	Hyperechoic band-like CaHA deposits; no acoustic shadowing	Superficial to extensor tendons	+++	None	None	5
50	F	3	Severe fat loss; moderate vein/tendon visibility	HA	1 mL injected	Elongated, hypoechoic, pseudocystic deposits	Superficial to extensor tendons	++	None	None	5
66	F	3	Severe fat loss; moderate vein/tendon visibility	PLLA+HA	PLLA: 1 vial in 8 mL sterile water + 2 mL lidocaine; 5 mL injected; then HA 1 mL injected	Subtle increased echogenicity of subcutaneous layer; pseudocystic HA deposits; hypoechoic linear bands	Superficial to extensor tendons	++	None	None	4
62	F	3	Severe fat loss; moderate vein/tendon visibility	PLLA+HA	PLLA: 1 vial in 10 mL sterile water; 5 mL injected; then HA 1 mL injected	Increased echogenicity of subcutaneous layer; pseudocystic HA deposits; edema	Superficial to extensor tendons	+++	None	None	5
48	F	2	Moderate fat loss; mild vein/tendon visibility	CaHA (Radiesse®)	1.5 mL CaHA + 0.5 mL lidocaine + 1.0 mL NSS; 1.5 mL mixture injected	Hyperechoic band-like CaHA deposits; no acoustic shadowing	Superficial to extensor tendons	+++	None	None	5
44	F	2	Moderate fat loss; mild vein/tendon visibility	HA	1 mL injected	Elongated, hypoechoic, pseudocystic deposits	Superficial to extensor tendons	++	None	None	5
50	F	2	Moderate fat loss; mild vein/tendon visibility	CaHA–HA hybrid (HarmonyCa®)	1.25 mL injected	Hyperechoic band-like deposits; rounder than CaHA alone	Superficial to extensor tendons	++	None	None	5
60	F	4	Extreme fat loss; severe vein/tendon visibility	CaHA–HA hybrid (HarmonyCa®)	1.25 mL injected	Hyperechoic band-like deposits; rounder than CaHA alone	Superficial to extensor tendons	++	None	None	4
50	F	3	Severe fat loss; moderate vein/tendon visibility	CaHA–HA hybrid (HarmonyCa®)	1.25 mL injected	Hyperechoic band-like deposits; rounder than CaHA alone	Superficial to extensor tendons	++	None	None	5

## Discussion

Rejuvenation procedures of the dorsal hand have gained increasing importance in recent years because the hands are considered the second most revealing indicator of aging after the face [2]. These procedures can be performed with several dermal fillers or with autologous fat transfer, and both approaches are currently regarded as safe, well-tolerated, and associated with low complication rates when performed by experienced injectors [1].

The choice of product is generally individualized and depends on the injector’s preference, familiarity with the material, and the patient’s anatomical needs. In our series, the most frequently used filler was CaHA (Radiesse®), which is consistent with previous reports on hand rejuvenation [7].

Pure CaHA-based fillers have been widely used for dorsal hand rejuvenation and have demonstrated favorable aesthetic outcomes and biostimulatory effects [8-10].

Hybrid formulations combining CaHA and hyaluronic acid aim to provide immediate volumization through the hyaluronic acid component and longer-term collagen stimulation through CaHA. The use of premixed CaHA-hyaluronic acid products has been specifically evaluated in studies addressing hybrid fillers [11].

Similarly, another publication supports the use of PLLA for dorsal hand rejuvenation because it stimulates collagen production and progressively restores soft-tissue volume, leading to reduced tendon and vein visibility, improved dermal texture, and better overall skin quality [12].

Fat grafting was performed in only one of our patients, which likely reflects the more invasive nature of this technique and the need for an operative setting. In this case, a 45-year-old woman underwent liposculpture under general anesthesia, and the harvested autologous fat was processed and transferred to the dorsum of the hand as detailed in the Materials and Methods section. Advocates of autologous fat transfer emphasize its biocompatibility, potential for revascularization, and capacity to deliver larger volumes with long-lasting results [13]. In this context, a systematic review reported that both CaHA fillers and autologous fat grafting demonstrate favorable and sustained aesthetic outcomes, without evidence of clear superiority of one technique over the other. These findings suggest that both approaches represent valid options for hand rejuvenation when appropriately selected for the individual patient [7].

HRUD is widely considered the imaging modality of choice for anatomical evaluation of the dorsal hand because it offers excellent spatial resolution and real-time dynamic assessment [14,15]. Cadaveric and ultrasound studies have shown that the dorsal hand contains three adipose laminae (superficial, intermediate, and deep) separated by the superficial, intermediate, and deep dorsal fasciae. The intermediate dorsal lamina is traversed by dorsal veins and sensory nerves, whereas the deep lamina contains the extensor tendons. The superficial lamina is free of vessels and nerves and primarily serves a protective and cushioning function. More recent descriptions indicate that the intermediate lamina is further subdivided by the subvenous dorsal fascia, which separates the dorsal venous plexus from the cutaneous nerves and creates an anatomically safer plane for injection [14,16].

Based on this anatomical framework, the ideal placement plane for hand rejuvenation products is superficial to the subvenous fascia, which is the same principle applied to fat grafting in the dorsal hand. Some authors, however, have proposed that when a greater volumetric effect is needed, autologous fat can also be placed in the deep lamina between the metacarpal bones to enhance three-dimensional fullness [1,16]. In our study, preprocedure HRUD enabled clear visualization of the dorsal fascial layers and adipose laminae and provided an objective correlate to the Merz Hand Grading Scale, especially for documenting soft-tissue atrophy, tendon prominence, and venous plexus visibility.

Post-procedure HRUD confirmed the correct placement of the injected materials in all patients, as the fillers were consistently located superficial to the extensor tendons. This positioning contributed to the desired aesthetic outcome and likely reduced the risk of complications. During the procedure, the cannula was maintained above the venous plexus, which was supported by the absence of hematoma, ecchymosis, or vascular compromise on both ultrasound and clinical assessment. On postprocedure imaging, the injected material appeared to partially encircle the dorsal venous plexus; however, this finding was interpreted as secondary to restoration of previously lost subcutaneous volume rather than true perivascular deposition of filler.

We observed no immediate or delayed complications at the 2-month follow-up visit. This finding is consistent with published data reporting low complication rates for both dermal fillers and autologous fat transfer when an appropriate plane is chosen, and blunt cannulas are used [7,17]. In addition, HRUD allowed us to recognize the characteristic sonographic patterns of the different materials (hyperechoic “cloud-like” bands for CaHA, pseudocystic hypoechoic deposits for hyaluronic acid, diffuse echogenicity changes for hyaluronic acid-PLLA mixtures, and isoechoic elongated deposits for autologous fat), which aligned with descriptions in previous studies [18-20]. Doppler examination immediately after treatment showed transient increases in vascularization in all patients, which we attributed to procedure-related hyperemia and to the early inflammatory and reparative phase associated with biostimulatory fillers.

Study limitations

This study has several limitations that should be considered when interpreting the findings. The short post-treatment follow-up period of two months precludes assessment of long-term durability and delayed adverse events, which are particularly relevant for volumetric fillers and autologous fat grafting.

Additionally, the descriptive design and limited sample size inherent to this case series restrict generalizability. Treatment selection was based on real-world clinical decision-making rather than standardized or randomized allocation, which may introduce selection bias and limit reproducibility.

Finally, aesthetic outcomes were assessed using the MARS, an author-developed, non-validated instrument. As such, results derived from this scale should be interpreted as descriptive and exploratory.

Future studies with larger cohorts, longer follow-up, and validated outcome measures are warranted to confirm these preliminary observations.

## Conclusions

Hand rejuvenation procedures have become increasingly relevant in aesthetic medicine. In this small multicenter case series, our findings provide preliminary and descriptive observations suggesting that HRUD may represent a valuable imaging tool for pre- and post-procedural assessment. HRUD allowed descriptive evaluation of dorsal hand anatomy, aging-related changes, and confirmation of filler or fat graft placement within the intended plane. These observations should be interpreted as exploratory and hypothesis-generating.

Although no definitive conclusions can be drawn, HRUD may facilitate early detection of potential complications and support a more objective and reproducible documentation of aesthetic outcomes, potentially enhancing procedural safety and precision in experienced hands. Further studies with larger, controlled cohorts and long-term follow-up are warranted to validate these preliminary observations, establish standardized imaging protocols, and better define the role of HRUD in supporting technique assessment and procedural planning in hand rejuvenation procedures.
